# A pilot study on the nanoscale properties of bone tissue near lacunae in fracturing women

**DOI:** 10.1016/j.bonr.2022.101604

**Published:** 2022-07-16

**Authors:** Wen Qian, Roman Schmidt, Joseph A. Turner, Sue P. Bare, Joan M. Lappe, Robert R. Recker, Mohammed P. Akhter

**Affiliations:** a Department of Mechanical and Materials Engineering, University of Nebraska-Lincoln, Lincoln, NE 68588-0526, United States of America; b Osteoporosis Research Center, Creighton University School of Medicine, Omaha, NE 68178, United States of America

**Keywords:** Bone, Lacunar, AFM-IR, Localized mechanical property, Mineral, Matrix

## Abstract

The goal of this study is to investigate the causes of osteoporosis-related skeletal fragility in postmenopausal women. We hypothesize that bone fragility in these individuals is largely due to mineral, and/or intrinsic material properties in the osteocyte lacunar/peri-lacunar regions of bone tissue. Innovative measurements with nanoscale resolution, including scanning electron microscope (SEM), an atomic force microscope that is integrated with infrared spectroscopy (AFM-IR), and nanoindentation, were used to characterize osteocyte lacunar and peri-lacunar properties in bone biopsies from fracturing (Cases) and matched (Age, BMD), non-fracturing (Controls) postmenopausal healthy women. In the peri-lacunar space, the nanoindentation results show that the modulus and hardness of the Controls are lower than the Cases. The AFM-IR results conclusively show that the mineral matrix, maturity (peak) (except in outer/far regions in Controls) were greater in Controls than in Cases. Furthermore, these results indicate that while mineral-to-matrix area ratio tend to be greater, the mineral maturity and crystallinity peak ratio “near” lacunae is greater than at regions “far” or more distance from lacunae in the Controls only. Due to the heterogeneity of bone structure, additional measurements are needed to provide more convincing evidence of altered lacunar characteristics and changes in the peri-lacunar bone as mechanisms related to postmenopausal women and fragility. Such findings would motivate new osteocyte-targeted treatments to reduce fragility fracture risks in these groups.

## Highlights

• Material properties around the osteocyte lacunae in human bone biopsies are presented.
• Using nanoindentation/nanoIR, the properties in bone tissue from women are measured.
• Mineral and material strength are different between fracturing and non-fracturing women.

## 1 Introduction

Osteocytes play a major role in mechanotransduction and remodeling of bone tissue. It is hypothesized that osteocytes directly contribute to the bone remodeling process (Bonewald, 2011; Seeman and Delmas, 2006; Dole et al., 2017; Roschger et al., 2019; Mullender et al., 2004). Specifically, the cellular network between osteocytes has been observed to sense local mechanical strains on the bone matrix and to receive signals that control bone matrix production, mineralization, and resorption. This sequence ties directly to recent studies testing the hypothesis that postmenopausal women who have sustained osteoporotic fractures have reduced bone quality, as indicated by measures of intrinsic material properties, in comparison with non-fracturing women (Vennin et al., 2017; Rokidi et al., 2019; Boskey et al., 2016). Lower lacunar density/volume would render low bone tissue porosity, causing bone tissue to have decreased toughness or energy absorbing ability thus compromising bone's capability to arrest cracks, leaving it more prone to fracture (Qiu et al., 2003; Heveran et al., 2019; Misof et al., 2019). Measurement of osteocyte lacunar properties presents numerous technical challenges. Due to the small size of individual lacuna, studying their volume, density, shape, and distribution is very challenging. Most techniques used to investigate the structure of bones include the scanning electron microscope (SEM) (Boyde, 2012), the transmission electron microscope (TEM) (Rubin et al., 2004), the confocal microscope (McCreadie et al., 2004; van Hove et al., 2009), and the use of Raman spectroscopy (Schrof et al., 2014). Although these techniques can locate and visualize osteocytes, they have significant limitations in terms of radiation damage and their inability to quantify mineral changes of the lacunae at the nanoscale.

Fourier transform infrared spectroscopy (FTIR) has been used to establish bone material quality: mineral-to-matrix ratio, crystallinity (i.e., mineral crystal size), carbonate-to-phosphate ratio (i.e., mineral maturity and crystallinity ratio), and collagen maturity (i.e., the level of enzymatic cross-linking) (Boskey et al., 2016; Boskey et al., 2009; Farlay et al., 2010; Schmidt et al., 2017; Gu et al., 2013; Unal and Akkus, 2015). The amide I band possesses structural information about the collagen matrix and is also the location of the strongest peaks for the non-enzymatic cross-link pentosidine. Therefore, the amide I peak was targeted here (1720–1600 cm^−1^) to find a sub-band ratio to quantify the effect of changes in the non-enzymatic cross-link content on the bone matrix. The mineral-to-matrix ratio, which characterizes the degree of mineralization of the bone matrix, is defined as the ratio of intensities of the phosphate stretch band to the amide I band. The crystallinity ratio is defined as the normalized intensity based on the values at 1030 cm^−1^/1110 cm^−1^. This ratio gives an index of mineral maturity and crystallinity corresponding to the transformation of nonapatitic domains to the apatitic ones. However, a complex biological tissue such as bone contains not only type I collagen fibrils, but also mineral and proteoglycan components. Therefore, additional tools are needed to discover the underlying organization of bone tissue. The atomic force microscope that is integrated with infrared spectroscopy (AFM-IR) is a rapidly emerging technique that provides chemical analysis with nanoscale spatial resolution that is far beyond conventional optical diffraction limits (Dazzi et al., 2012). AFM-IR uses an AFM contact probe to detect the local thermal expansion in a sample which results from absorption of infrared radiation during a measurement (Dazzi and Prater, 2017). Such an approach can also be used to map the chemical response spatially in bone tissue (Dazzi and Prater, 2017).

Since its introduction, instrumented indentation testing (IIT) or nanoindentation has been widely used to measure the elastic modulus and hardness of the peri-lacunar space and bone lamellae at submicron scales, but challenges remain with respect to separation of the contributions from the collagen and mineral (Hengsberger et al., 2002; Saini et al., 2019). Although, the mineral/matrix composition properties (Rokidi et al., 2019; Rizzo et al., 2018; Mandair et al., 2021) and local intrinsic/micromechanical properties (Akhter et al., 2017) have been reported in these biopsies, the properties have been localized within the peri-lacunar space in animal models (CTI, 2020) (Taylor et al., 2020), but not in human biopsies before. Bone receives stress (external force) which produces strain (structural deformation). Higher level strains above the yield point deform bone material beyond its point of resilience (JMNI) (Nicolella et al., 2008), consequently generating material damage, usually in the form of micro-cracks. It has been reported that intrinsic material properties may affect the local lacunar mechanical strains during skeletal loading events (Nyman et al., 2011). The osteocytes within lacunae are sensitive to mechanical strain and help regulate bone remodeling and other functions. It is expected that the lacunar (peri-lacunar) mineral/matrix properties are correlated with the intrinsic bone quality, independent of the bone mineral density (BMD), in comparisons of fracturing and matched, non-fracturing postmenopausal women (Dole et al., 2017; Vennin et al., 2017; Akhter et al., 2017). It was also observed that osteocyte lacunae are larger, more numerous and more spherical in healthy women (Bonewald, 2011; Qiu et al., 2003). An understanding of the organization of mineral and matrix within the peri-lacunar space along with information about the mechanical properties of each constituent are needed to understand initiation and to quantify the propagation of fracture in bone tissue (Yeni et al., 2001). In this manuscript, we combine three different approaches including SEM, AFM-IR, and nanoindentation, to characterize osteocyte lacunar and peri-lacunar properties in bone biopsies from fracturing (n = 5, Cases) and matched (Age, BMD), non-fracturing (n = 5, Controls) healthy postmenopausal women. Based on published data, it is possible that osteocyte lacunar/peri-lacunar properties play a role in the local or intrinsic properties of bone tissue thus affecting its material strength (CTI, 2011) (Taylor et al., 2020), We hypothesize that bone fragility in fracturing individuals is largely due to mineral, and/or intrinsic material strength that include osteocyte lacunar/peri-lacunar properties (Frost, 1960a; Frost, 1960b; Arnold et al., 1971). To the best of our knowledge, this is the first study to investigate the causes of osteoporosis-related skeletal fragility in fracturing postmenopausal women that considers the mechanical behavior with respect to relative distance from the lacunae.

## 2 Methods

### 2.1 Position registry

The mineral organization and properties in the peri-lacunar region were quantified using three different experimental techniques, including SEM/AFM-IR/nanoindentation, all with nanoscale spatial resolution. These regions of interest were within ~25 μm of the lacuna center. The goal was to ensure that the same lacunae could be identified/located after the samples were moved from instrument to instrument (SEM to AFM-IR, to IIT), so that the results from all instruments could be correlated spatially. First, each lacuna of interest was identified with SEM within a bone biopsy sample. The position of each lacuna was recorded relative to the specific shape of the central canal as well as the surrounding cement lines as fiducial markers. Next, the mineral organization measurements were made using AFM-IR (Sereda et al., 2019). Using the fiducial markers, each lacuna was identified with an optical microscope and the probe positioned accordingly in the peri-lacunar space. Localized nanoIR spectra were acquired at several positions in this region and relevant nanoIR absorption peaks were collected. Finally, the samples were tested for their nanoscale mechanical properties of elastic modulus and hardness in the peri-lacunar region. Again, the specific fiducial markers were used to identify the target lacuna in order to position the IIT tip in the regions of interest.

### 2.2 Bone sample preparation

A longitudinal section of 300 μm in thickness was cut from each bone biopsy that included trabecular and cortical bone (Akhter et al., 2017; MPLJ, 2019). Each biopsy section was mounted on a glass slide, and polished to 0.3 μm surface finish required for subsequent measurements. These sections were obtained from PMMA-embedded biopsy blocks prepared for histological investigations in a prior study (Boskey et al., 2016). All measurements were performed around the edge of each lacuna, which assured that the measured spots were mineralized bone. Embedding bone typically increases the variance of most peak ratios, but differences in the compositional properties were still detectable in samples embedded in PMMA (CTI, 2011) (Nyman et al., 2011). In each biopsy, the investigation was focused on cortical bone. The sub-groups of biopsies in this study represent fracturing (n = 5), and non-fracturing (n = 5) postmenopausal women. The bone biopsy details have been published elsewhere (Vennin et al., 2017; Rokidi et al., 2019; Boskey et al., 2016; Rizzo et al., 2018; Mandair et al., 2021; Kimmel et al., 2022). The two groups of healthy, non-osteoporotic postmenopausal women who were at least 4 years past their last menstrual period were recruited, including a) fragility fracture women and b) BMD/age-matched women with no fragility fracture.

At the time of enrollment, the fragility fracture subjects were healthy postmenopausal women who experienced fracture during the previous five years due to low trauma and had never taken any type of bone-active medication. “Low trauma” was defined as trauma equal to or less than a fall to the ground from standing height. Fractures of digits, face, and skull were excluded. The fracture group (Cases) included post-menopausal women with osteopenic BMD values (T-scores between +0.3 and −2.5 for either the hip or spine) (Vennin et al., 2017; Rokidi et al., 2019; Boskey et al., 2016; Rizzo et al., 2018; Mandair et al., 2021; Kimmel et al., 2022).

### 2.3 Localized nanoIR spectrum for mineral and matrix characterization

**Fig. 1:**
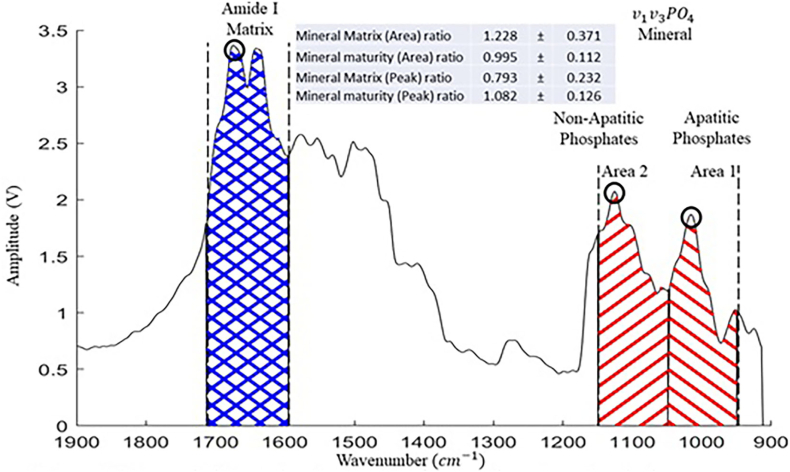
Schematic illustration (example) of area ratio and peak ratio for a localized infrared spectrum of a Case bone biopsy specimen (Mean ± Std).

For bone compositional analysis, mineral and matrix characterization via AFM-IR was performed (Sereda et al., 2019). A commercial nanoIR2 atomic force microscope (Anasys Instruments, Inc.) supplemented with a tunable infrared quantum cascade laser, QCL (Daylight Solutions MIRcat), was used to image topography and measure localized nanoIR spectra, as well as to create chemical IR maps at a constant wavelength. Contact mode nIR2 probes (Model: PR-EX-nIR2, Anasys Instruments) with resonance frequency of 13 ± 4 kHz and spring constant of 0.07–0.4 N/m were used. The AFM-IR instrument has a pulsed tunable IR source, with a pulse length of ∼10 ns and can cover a broad range of the mid-IR region. The light from the IR laser was focused onto the surface region near the tip-sample contact area. When the sample absorbed the light, a rapid heating/expansion of the sample occurred which created an impulsive load onto the AFM cantilever tip that induced an oscillation. The amplitude of cantilever oscillation was proportional to the sample IR absorption coefficient. Spectra were acquired with two perpendicular directions of infrared laser light. Peak amplitude normalization was applied to all spectra in order to visualize and compare spectral shape/peak ratios. Spectra were acquired over a range of 1900 to 912 cm^−1^. Each spectrum was acquired two times, automatically averaged for better signal to noise ratio and the background noise was subtracted using the instrument software. Each individual spectrum was fit with Gaussian functions using a customized MATLAB code (MathWorks, Inc.) and was treated as a single point. Mineral-to-matrix ratio was calculated by dividing the area of the phosphate peak (1150–950 cm^−1^) by the area of the amide I peak (1720–1600 cm^−1^). Matrix maturity ratio was calculated by dividing the apatitic phosphate peak area (1050–950 cm^−1^) by the non-apatitic phosphate peak area (1150–1050 cm^−1^). The definitions used for the calculations are shown schematically in Fig. 1. These values were used for comparisons between the Controls and Cases. A total of 208 nanoIR spectra were collected on a total of sixteen lacunae, six lacunae from the Controls, ten lacunae from the Cases.

### 2.4 Nanoindentation testing of mechanical properties

The localized mechanical properties (modulus and hardness) of the peri-lacunar region were measured using a Hysitron TI 950 Triboindenter through quasistatic nanoindentation (Vennin et al., 2017). A Berkovich (three-sided pyramid) diamond tip was mounted on a transducer that allowed for displacements in the z direction. The bone sample was mounted on a scanner that allowed for motion in the x/y-plane that was perpendicular to the tip axis. This combination allowed the topography of the sample surface to be mapped, using the scanning probe microscope (SPM) mode, and force-displacement measurements to be made using the same tip. Based on the SPM image, the tip was positioned on the peri-lacunar region to quantify mechanical differences with respect to position. During an indentation measurement, the tip was pressed into the sample such that the resulting force-displacement behavior was quantified. Multiple indentations were made with a target force of 6 mN (milliNewtons) at a constant loading rate of 400 μN/s (microNewtons per second) (Vennin et al., 2017). The indentation procedure included a linear loading ramp of 15 s, a holding period of 10 s at the maximum load and a linear unloading ramp of 15 s. The load-displacement data from each indentation were used to calculate the reduced indentation modulus (Er) and hardness (H) for the tissue. Mechanical measurements of modulus and hardness were made with ~10 μm spacing, in order to identify differences of the volume fraction of mineral near the lacunae. A Poisson's ratio of 0.3 was assumed for bone tissue in calculations for the analysis (Hengsberger et al., 2002; Reilly et al., 1974; Zysset et al., 1999). The Oliver-Pharr method (Oliver and Pharr, 1992) was used to extract the indentation modulus. A total of 315 nanoindentation results were collected on the total of twenty-eight lacunae, including fourteen lacunae from Control samples, and fourteen lacunae from Case samples.

### 2.5 Morphology of lacunae

The morphology of the bone and osteocyte lacunae (Akhter et al., 2017; MPLJ, 2019) were verified and observed using a field-emission scanning electron microscope (FESEM, FEI Helios Nanolab 660). Furthermore, the localized AFM-IR spectra of the peri-lacunar space in the vicinity of ~25 μm around the lacunae were collected. A total of 14 to 16 positions were collected for each peri-lacunar space and both “lacunae-near” and “lacunae-far” were analyzed (approximately n = 30 measurements/positions or points per specimen) for Controls (Fig. 4a-b) and Cases (Fig. 4c-d) respectively. The position of “lacunae-near” was within the 5 μm distance from each lacunar edge. The position of “lacunae-far” was within the 15 μm distance from each lacunar edge. The distance between “near” and “far” was around 10 μm. Mineral-to-matrix ratio represents the degree of mineralization of the bone tissue and the areas under the curve of the phosphate peak (1140–912 cm^−1^) and the amide I band (1720–1600 cm^−1^) were integrated to measure the mineral-to-matrix area ratio, as shown in Fig. 1. Mineral maturity and crystallinity ratio corresponds to the progressive transformation of immature surface-hydrated domains into a mature and more stable apatite lattice. Thus, the areas under the curve of the phosphate peak (1050–912 cm^−1^) and the peak (1140–1050 cm^−1^) were integrated to define the mineral maturity and crystallinity area ratio. The collected localized IR spectra were used to assess the bone quality quantitatively through normalization of IR absorption peaks, by analyzing the values of “lacunae-far” (Fig. 4a, Fig. 4c) vs “lacunae-near” (Fig. 4b, Fig. 4d) in Control and Case patients.

### 2.6 Statistics analysis

nanoIR spectral statistics were performed using IBM SPSS-v23. In a standard normal distribution, the negative twenty fifth percentile and the positive seventy fifth percentile have a Z score |0.67| with an interquartile range of 1.34. Five times the interquartile range, added to the seventy fifth percentile, will provide a range of acceptable data. To reiterate, any data with a Z score larger than 2.68 or lower than −2.68 can safely be deemed as outliers. A total of 9 (4 % approximately) such data points were identified from the original 208 data points. A Matlab program was used to identify and systematically remove all the statistical outliers or noise in the nanoIR data. From each set of testable data, all the variables (Table 2) were compared between the Cases and Controls using the Wilcoxon signed rank test to compare their medians (P < 0.05). All lacunar data are presented as medians and interquartile range (Table 2). Intrasample variance was calculated (coefficient of variation = standard deviation/mean for the multiple measurements of cortical bone tissue).

## 3 Results

**Fig. 2:**
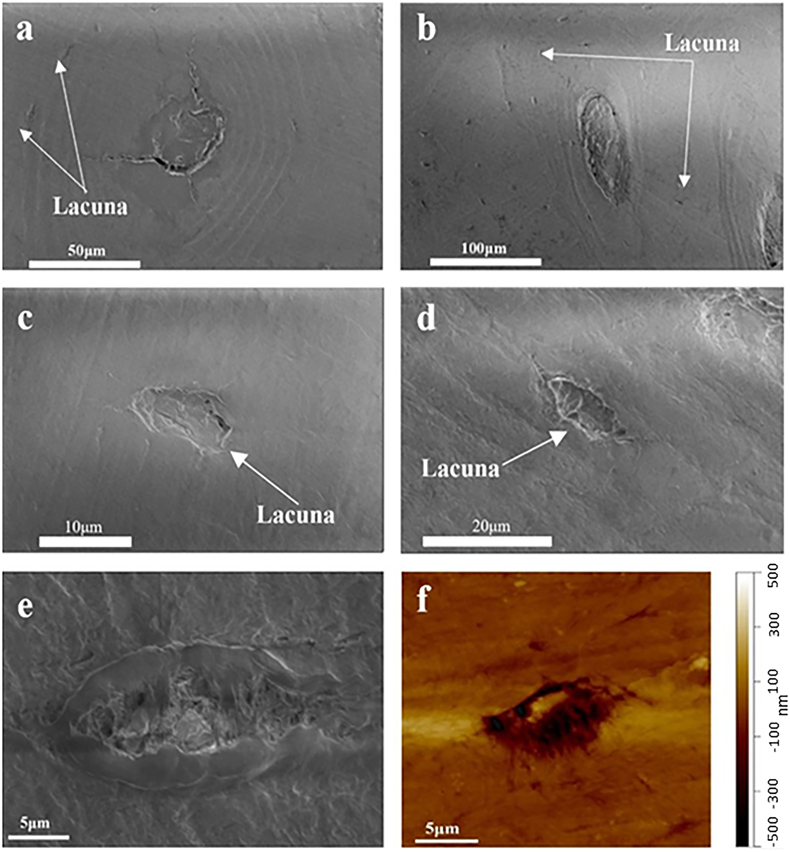
SEM images of a central canal, with surrounding cement lines and lacuna for Control (a) and Case (b) samples respectively; Enlarged SEM images of lacunae /peri-lacunae regions within the cortical bone tissue for Control (c) and Case (d) samples respectively; Enlarged SEM image (e, control), and AFM topography image (f, case) of lacunae showing the mineralized fibrils.

SEM imaging was used to identify a region of interest within each sample. In Fig. 2, SEM images show an osteon with a haversian canal at the center which is surrounded by concentric lamellae of collagen fibers, for the Control (Fig. 2a) and the Case (Fig. 2b) respectively. Both the lacunar/peri-lacunar regions within the cortical bone tissue for the Control (Fig. 2c) and the Case (Fig. 2d) were identified. As noted previously, the lacunae in cortical bone are more rod-like in shape than those in trabecular bone (Akhter et al., 2017; MPLJ, 2019). This result may suggest indirectly that cortical bone osteocyte lacunae are better adapted for interception of propagating micro-cracks that could eventually lead to micro-damage accumulation and ultimately stress fracture (Busse et al., 2010a). Fig. 2-e,f shows the SEM image and AFM topography image of lacunae with mineralized fibrils in the lacunar void, which consists of nanoscale collagen and minerals.

**Fig. 3:**
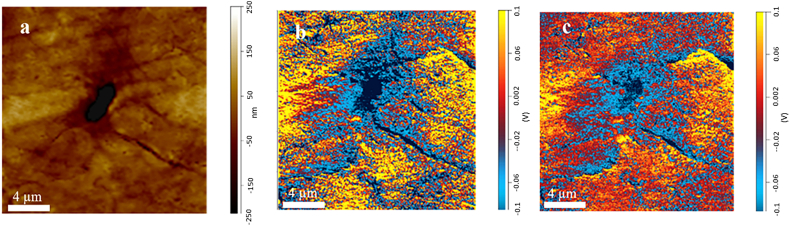
AFM topography image of regions around the lacunae (a), the corresponding chemical mapping of matrix at 1655 cm^−1^ (b) and at mineral 1015 cm^−1^ (c) respectively.

Fig. 3 shows the AFM topography (Fig. 3a) and the corresponding chemical mapping of mineral (Fig. 3b) and matrix (Fig. 3c) around the lacunae respectively, which reveals the spatial variation of mineral arrangement which can be related to mineral-to-protein (collagen) or carbonate distributions. These results demonstrate the power of AFM-IR to map naturally formed mineralized nanostructures. Through chemical IR mapping, mineral precipitation, aggregation, and aging can be analyzed and quantified at the nanoscale, to provide new understanding of the biological processes of lacunae formation.

**Fig. 4:**
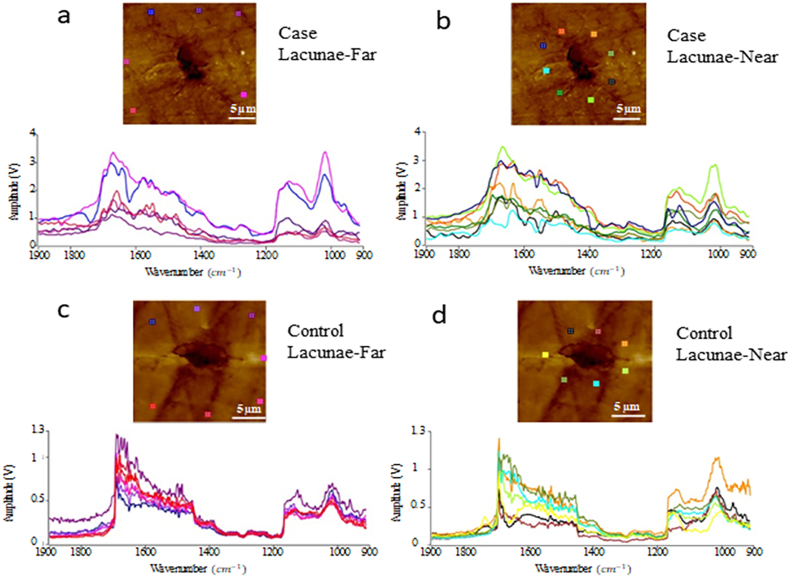
Comparison of Localized IR spectrum between “lacunae-far” and “lacunae-near” for Case (a-b) and Control (c-d) samples respectively.

Fig. 4 shows the comparison of localized IR spectra between “lacunae-far” and “lacunae-near” for one pair of Case and Control samples respectively. The IR spectra were fit, and the mineral-to-matrix ratio and mineral maturity and crystallinity ratio of distance related lacunae were extracted and are summarized in Table 1. Both ratios for “lacunae-near” are higher than those for “lacunae-far”, which is probably due to relative-filling of the mineral in the lacunar void (Boskey et al., 2009; Frost, 1960a; Frost, 1960b; Arnold et al., 1971; MPLJ, 2019).

**Table 1:** Localized infrared property of distance related lacunar in Control and Case pairs bone samples. These data reflect a pair of samples (Case and Control) showing mineral/matrix (MM) and mineral maturity and crystallinity (mmc) distribution around lacunae (Mean ± Std).

Type	MM area ratio	mmc area ratio	MM peak ratio	mmc peak ratio
Control Lacuna-Near	1.500 ± 0.575	1.305 ± 0.284	0.710 ± 0.285	1.371 ± 0.252
Control Lacuna-Far	1.191 ± 0.243	1.128 ± 0.085	0.593 ± 0.130	1.310 ± 0.166
Case Lacuna-Near	1.032 ± 0.329	1.061 ± 0.077	0.696 ± 0.237	1.231 ± 0.209
Case Lacuna-Far	0.913 ± 0.352	1.019 ± 0.155	0.652 ± 0.272	1.193 ± 0.289

**Fig. 5:**
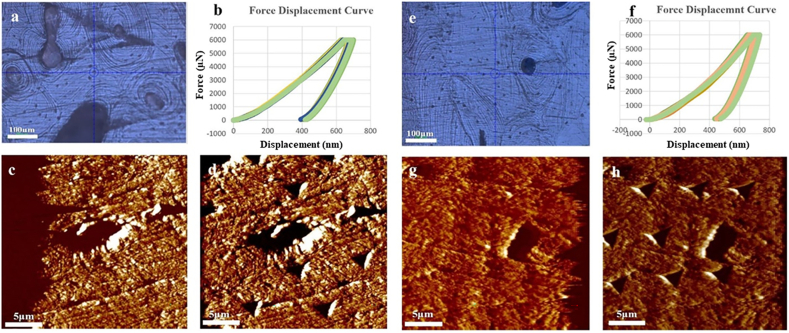
Nanoindentation of Case sample (a-d) and Control sample (e-h). (a) Optical microscope image (10×) of cortical bone; (b) a total of nine force-displacement curves around the lacunae; (c-d) Topography image of lacunae before (c) and after (d) indents respectively; (e) Optical microscope image (10×) of cortical bone; (f) a total of nine force-displacement curves around the lacunae; (g-h) Topography image of lacunae before (g) and after (h) indents respectively.

Fig. 5a and Fig. 5e shows the optical image of Case sample (5a) and Control sample (5e) of cortical bone from the IIT instrument. A total of nine quasi-static indentations were performed within the peri-lacunar space (18 indents for two lacunae per specimen) on the cortical bone (Fig. 5b Case and Fig. 5f Control), to acquire the elastic modulus and hardness, in order to quantify differences of the mineral near the lacunae. Regions of lacunae area were first identified using SPM imaging to quantify the surface topography (Vennin et al., 2017). After imaging, the indent positions were chosen around the lacunae. Fig. 5c-d and Fig. 5g-h shows the SPM topography images with scan size of 27 μm by 27 μm before (Fig. 5c and e) and after (Fig. 5d and h) indentations for Case and Control respectively. The triangular imprints show the measurement locations and their size. The descriptive statistics and the differences for the nanoindentation data set are shown in Table 2. The median hardness value (0.595 GPa) for Controls was lower than Cases (0.809 GPa), and modulus values for Controls (12.42 GPa) are also lower than Cases (13.29 GPa). The variance (Table 2) of the measurements for most of the variables declined in Controls compared to Cases.

**Table 2:** Osteocyte lacunar properties in fracturing and non-fracturing women of five pairs of samples. The position of lacunae-near was within the 5 μm distance from each lacunar edge. The distance between “near” and “far” was around 10 μm. (Mean ± Std).

	Control Median (IQ^d^ range)	Case Median (IQ^d^ range)
Modulus	12.42 (5.092–21.95)	13.29 (9.88–23.19) a
Modulus variance	0.195 (0.095–0.621)	0.240(0.085–0.699)
Hardness	0.595 (0.241–0.893)	0.809 (0.563–0.941) a
Hardness variance	0.241(0.102–0.700)	0.229(0.112–0.414)
Lacunae-near		
Mineral matrix (area)	1.303 (0.997–1.601) c	1.056 (0.845–1.250) a
Mineral matrix variance (area)	0.188(0.141–0.379)	0.267(0.162–0.304)
Mineral maturity and crystallinity (area)	1.084 (0.892–1.160)	0.848 (0.769–1.055) a
Mineral maturity and crystallinity Variance (area)	0.076 (0.051–0.164)	0.094 (0.064–0.165)
Mineral matrix (peak)	0.754 (0.630–1.048)	0.651 (0.4916593–0.831) a
Mineral matrix variance (peak)	0.180(0.132–0.405)	0.251(0.167–0.337)
Mineral maturity and crystallinity (peak)	1.233 (0.999–1.384) b	1.010 (0.857–1.145) a
Mineral maturity and crystallinity Variance (peak)	0. 054(0.046–0.114)	0.127(0.099–0.186) a
Lacunae-far		
Mineral matrix (area)	1.196 (0.889–1.460)	1.062 (0.859–1.335)
Mineral matrix variance (area)	0.233(0.153–0.317)	0.179(0.138–0.033)
Mineral maturity and crystallinity (area)	1.075 (0.872–1.205)	0.827 (0.772–1.021) a
Mineral maturity and crystallinity Variance (area)	0.078(0.059–0.199)	0.104(0.083–0.151)
Mineral matrix (peak)	0.711 (0.554–0.859)	0.667 (0.476–0.952)
Mineral matrix variance (peak)	0.262(0.153–0.494)	0.205(0.137–0.305)
Mineral maturity and crystallinity (peak)	1.214 (0.987–1.356)	0.952(0.833–1.063) a
Mineral maturity and crystallinity variance (peak)	0.112(0.088–0.137)	0.138(0.072–0.222)

Mean ± std.

a Difference between Case and Controls, P < 0.05.

b Difference between “near” and “far” lacunae (within each group), P < 0.05.

c Difference between “near” and “far” lacunae (within each group), P < 0.1.

d IQ-interquartile range (25th percentile – 75th percentile).

The data acquired (both “near” and “far” positions) of the mineral to matrix ratio (area) and mineral to matrix ratio (peak) show that the values for the Control are significantly higher than the Cases (Table 2, only the “lacunar-far” position mineral matrix (area) and mineral matrix (peak) not different), which agrees with most of the research elsewhere (Vennin et al., 2017; Misof et al., 2019). However, the standard deviation or variance (Table 2) for the Controls is 1.1–1.6 times that of the Cases which suggests more variations in the mineral/material properties of non-fracturing subjects (Busse et al., 2010a; Gerbaix et al., 2017). Furthermore, AFM-IR results indicated that while the mineral-to-matrix area ratio tended to be greater (P < 0.1), the mineral maturity and crystallinity peak ratio “near” lacunae are greater (P < 0.05) than at regions “far” or more distance from lacunae in the Controls only (Vennin et al., 2017; Rokidi et al., 2019; Boskey et al., 2016).

### 3.1 Correlation between minerals volume and localized mechanical property

**Fig. 6:**
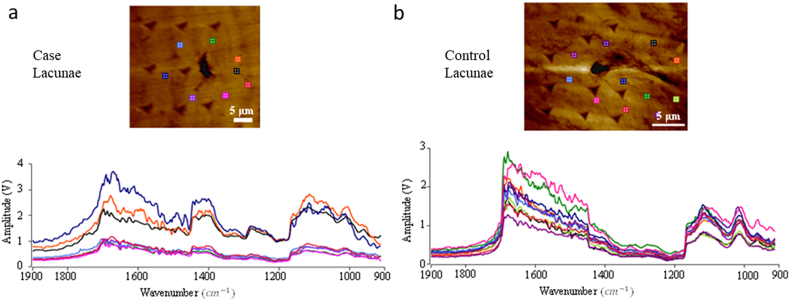
The localized IR spectrum and nanoindentation testing around the same lacunae for Case (a) and Control (b) respectively.

Fig. 6 shows the localized IR spectra and nanoindentation results around the same lacunae for one pair of Case (6a) and Control (6b) respectively. Table 3 shows the localized infrared results of lacunae and corresponding mechanical properties in one pair of Control and Case bone samples. No significant relation between the mineral composition (mineral/matrix, mineral/maturity ratio) and the local indentation modulus/hardness within the bone tissue of Control and Case is identified.

**Table 3:** Localized infrared property of lacunar and corresponding mechanical properties in Control and Case pairs bone samples, mineral matrix ratio (MM ratio), mineral maturity and crystallinity ratio (mmc ratio). These data represent both mechanical and mineral property (infrared spectra) distribution within a pair of specimens (Case and Control) (Mean ± Std).

Type	MM area ratio	mmc area ratio	MM peak ratio	mmc peak ratio	Modulus (GPa)	Hardness (GPa)
Case	1.379 ± 0.272	0.787 ± 0.017	0.783 ± 0.173	0.856 ± 0.032	15.468 ± 0.697	0.775 ± 0.076
Control	1.168 ± 0.267	0.919 ± 0.078	0.697 + 0.166	1.08 ± 0.095	11.615 ± 0.859	0.680 ± 0.073

## 4 Discussion

The objective of this study was to provide new insights into bone fragility in postmenopausal women by analyzing local or intrinsic material properties of bone tissue. We hypothesize that the skeletal fragility is due to mineral, and/or intrinsic material properties that can be identified in the osteocyte lacunar/peri-lacunar region of the bone tissue. Our results suggest there is merit to this concept.

### 4.1 Peri-lacunar mechanical properties

The peri-lacunar mechanical properties (Table 2) are different than the interstitial bone tissue data published from these biopsies, reported by Vennin et al. (2017). The indentation modulus of Controls in the peri-lacunae is less than the interstitial bone, but the modulus and hardness of Cases in the peri-lacunar region are higher than interstitial bone tissue (Vennin et al., 2017). The mineral exchange in lacunar voids is a specific process, sometimes called mineral diffusion and micropetrosis. This mechanism of mineral accumulation, during micropetrosis in lacunae, may be the cause of the higher hardness and modulus in Cases (Table 2) and thus may be responsible for a reduction in toughness (more brittleness) despite a lower mineral/matrix ratio. Furthermore, Rokidi et al. (2019) reported lower nano-porosity and tissue water in Cases -another contributing factor towards bone fragility. A similar process of mineral-filling (micropetrosis) contributing to modify the lacunar spherical shape, by reducing the size/volume, and increasing the fragility, which was reported in bone tissue from a mouse animal model when subjected to 4wks of space flight (Gerbaix et al., 2017). These data (Gerbaix et al., 2017) document the unique role of the mineral/matrix ratio of bone tissue quantified at the peri-lacunar indentation sites, suggesting an inverse relation between the intrinsic strength and mineral/matrix ratio.

### 4.2 Mineral properties

Additional investigation of the mineral type (amorphous or crystalline) may further explain this inverse relationship (between strength and mineral/matrix ratio) (Gerbaix et al., 2017) in the peri-lacunar space, regardless of the position or distance from the lacunae (Table 1, Table 2, Fig. 4). The increase in mineral properties near the lacunar edge suggests continued filling with mineral only, while the matrix remains relatively unchanged as suggested previously (Frost, 1960a; Frost, 1960b; Arnold et al., 1971; Remaggi et al., 1998). The variation in mineral properties within the peri-lacunar regions that are linked to bone fragility along with the mineral property changes that are directly related to bone tissue mechanical properties should be further investigated (Busse et al., 2009). The mineral composition variation between two measured points (near and far) within the vicinity of the peri-lacunar space represents heterogeneity/variation within both the Cases and the Controls. The greater trends of variation in modulus and hardness within Cases (Table 2) may represent continued filling of the lacunar spaces due to osteocyte apoptosis (Misof et al., 2019; Milovanovic et al., 2017) causing shrinking of lacunar volume (Bell et al., 2008). However, the active mineral exchange in a live healthy osteocyte lacunar space may reflect different nanomechanical properties (Busse et al., 2010a; Gerbaix et al., 2017) inducing a greater toughness in bone tissues within Controls.

### 4.3 Filling of lacunar voids-micropetrosis

Both Frost and Bell (Frost, 1960a; Bell et al., 2008) observed the mineral filling of the lacunar space along with the canalicular space with aging. Frost noticed an increase in brittleness even in the bone sections that exhibited greater micropetrosis (Frost, 1960b). Furthermore, in agreement with others (Qiu et al., 2003), micropetrosis may produce mineral filled, smaller in size, less numerous lacunae, causing bone tissue from the Cases to be harder and more brittle (Busse et al., 2010b; Milovanovic et al., 2017). Our high-resolution data on these specimens suggest that bone tissue from Cases has declining lacunar volume compared with the non-fracturing Controls (MPLJ, 2019; Akhter and Recker, 2021), concurring with the Frost (Frost, 1960a; Frost, 1960b) data with regards to micropetrosis in Cases. It is possible that with micropetrosis (Gerbaix et al., 2017), the mineral diffusion and exchange is minimal, therefore, the mineral is packed and aged resulting in greater indentation modulus/hardness properties in peri-lacunar space. On the other hand, in Controls, the healthy osteocytes continue to provide mineral exchange in peri-lacunar surface allowing it to be less likely to produce a tougher bone tissue (Table 2). Unlike previous studies of nanoindentation (Vennin et al., 2017; Ferguson et al., 2003), the measurements here targeted only the peri-lacunar region (within 5 μm of the lacunar edge—“near” and <15 μm distance from lacunar edge—“far”) to quantify mineral property differences between bone tissue from Cases (fracturing) and non-fracturing postmenopausal women (Controls). Please note while mineral matrix provides information on the mineral and matrix components, the mineral maturity and crystallinity reflects a progressive transformation to more stable apatite lattice in bone tissue (Farlay et al., 2010). Interestingly, in Controls only, the mineral/ maturity properties were greater at positions closer to the lacunar edge. Again, additional data are needed to identify the source of the variation (i.e., mineral type or maturity).

The filling of lacunae and the canalicular system (Rokidi et al., 2019; Frost, 1960c) with mineral reduces their size or volume (MPLJ, 2019) resulting in dried bone tissue (Boyde, 2012) with lower spatial heterogeneity (Rizzo et al., 2018), along with increased modulus/hardness (Table 2). In Cases, such a progression may be responsible for the reduction of both the intrinsic and extrinsic toughness (Lloyd et al., 2017). Decreased intrinsic toughness will allow the formation of microcracks at a relatively low stress, and extrinsic toughness will facilitate the growth of a microcrack, both of which contribute to the fragility of bone tissue (Gerbaix et al., 2017; You et al., 2001).

Although no correlation was found between the mineral composition and the local indentation modulus/hardness (Cases or Controls) for these 5 pair of specimens, the declining mineral matrix ratio was reported to be responsible for lower indentation modulus and hardness in trabeculae of osteoporotic vertebral bodies (Yao et al., 2011). However, others (McCreadie et al., 2004; McCreadie et al., 2006; Ferretti et al., 1999; Tai et al., 2005; Oyen et al., 2008; Zebaze et al., 2011; Lloyd et al., 2015; Heveran et al., 2019) showed no clear relationship between mineral composition and indentation modulus in fracturing Cases and Controls. The results presented here may suggest that within the proximity of lacunar voids, the mineral/matrix properties are different than values reported previously from the interstitial region (Vennin et al., 2017). Furthermore, along with mineral properties, the stiffening of the collagen matrix could also be responsible for fractures. Remaggi et al. (Ferretti et al., 1999) reported that mineral diffusion is a primary effect as opposed to matrix modification in the canalicular and lacunae system, thus providing unique micro-level strength difference from the interstitial bone tissue (still under investigation).

### 4.4 Novel combination of nanoIR & nanoindentation to quantify local properties in peri-lacunar space

Our nanoIR spectra and nanoindentation measurements on the same lacunae (Fig. 6) show no significant relationship between mineral matrix, mineral maturity and crystallinity, and mechanical property variables (Table 3). These results suggest that the observed differences in the intrinsic mechanical properties of the two areas (within the peri-lacunar space) may not be attributed to local bone mineral properties. The real mechanism for the mineral accumulation or exchange in canalicular/perilacunar space is not well known (Oyen et al., 2008). This study is the first to provide data with regards to mineral and the corresponding local nanomechanical properties within the peri-lacunar space. Although the peri-lacunar space is proposed to have predominantly mineral (Oyen et al., 2008), the mineral properties need to be explored more extensively. The mineral density (packing) in the peri-lacunar space for the shrinking lacunae due to micropetrosis in Cases (Yao et al., 2011; Oyen et al., 2008) may be different than the properties from the healthy and larger osteocyte lacunae in Controls (Oyen et al., 2008).

### 4.5 Variations in the properties

The variances (modulus and hardness, Table 2) suggests that the bone tissue in the peri-lacunar regions of Controls had less heterogeneity in comparison with Cases. A more detailed interpretation of these mechanical data requires a better understanding of the intrinsic mechanical properties of the bone matrix constituents and, especially, the strength of the bonds between the organic and mineral components. An improved understanding of bone mechanics is vital for the development of evaluation strategies for patients at risk of bone fracture. Bone matrix material properties depend on the hierarchical structural features that make up bone as well as their composition. The origin of these properties and their dependence upon the hierarchical structure and composition of bone tissue need additional investigation.

The difference in mineral properties between Controls and Cases is most likely due to the complexity of biological tissue and the natural variation in mineral heterogeneity in the bone biopsy samples.

It is proposed here that these nanoscale results provide additional detailed information of bone tissue properties and provide context to the microscale and macroscale data acquired previously (Vennin et al., 2017; Rokidi et al., 2019; Boskey et al., 2016). Please note that by making measurements at approximately 10 specific positions around a lacuna (~25 μm apart) the data are expected to reflect the realistic variations of the bone tissue with respect to the region of interest.

## 5 Limitations

These bone biopsies do not represent actual fracture sites. Therefore, the data presented here may not represent the actual mineral and nanomechanical properties experienced near relevant fractures. However, each biopsy allows a unique window into the bone health status with respect to the intrinsic material properties which may be a surrogate to skeletal health. All the biopsies were subject to standard and equivalent embedding procedure and therefore we expect minimal noise related to their embedding. Nonetheless, the iliac crest is the standard anatomical site for obtaining bone biopsies, and these unique data in the peri-lacunar space were collected using three different techniques in the same local region. Furthermore, these data were from a small number of specimens randomly selected from a larger set reported previously (Vennin et al., 2017; Rokidi et al., 2019; Boskey et al., 2016).

## 6 Conclusions

In summary, this is the first study to investigate the osteocyte lacunar/peri-lacunar properties of bone tissue in fracturing (Case) and non-fracturing (Control) postmenopausal women. The unique combination of SEM, AFM-IR, and nanoindentation were used to investigate a direct relationship between lacunar properties and intrinsic mechanical properties. The nanoindentation results show that both hardness and indentation modulus of peri-lacunar space in the cortical bone were greater in Cases than Controls. In addition, an increase in material heterogeneity (variance in the hardness and modulus was observed in the Controls as compared with the Cases. The AFM-IR results conclusively show that the mineral matrix, maturity (peak) (except in outer/far regions in Controls) were greater in Controls than in Cases. Furthermore, mineral maturity and crystallinity ratios of “lacunae-near” are on average higher than those from “lacunae-far” in Controls only. The complex relationship of spatial variation in material properties (mineral composition, local hardness/modulus, etc.) (Lloyd et al., 2015) within and around the peri-lacunar space would need additional data to explain fragility in bone tissue from fracturing individuals. Pending resources, the ongoing work will be expanded to include 120 pairs of samples to provide an additional extensive data analysis that is required to provide more convincing evidence of possible alterations in the lacunar characteristics and changes in the peri-lacunar bone as mechanisms related to postmenopausal women and fragility fractures. The findings would motivate new osteocyte-targeted treatments to reduce fragility fracture risks in postmenopausal women.

## Data availability

The raw/processed data required to reproduce these findings cannot be shared at this time as the data also forms part of an ongoing study.

## CRediT authorship contribution statement

All authors contributed equally in the preparation of this manuscript.

## Declaration of competing interest

All authors above have NO conflict of interest with regards to this submitted paper to Bone Journal.

## Appendix A Supplementary data

Supplementary material Image 1
