# Supplementary material for: A pilot study on the nanoscale properties of bone tissue near lacunae in fracturing women
Source: Bone Rep. 2022 Jul 16;17:101604. doi: 10.1016/j.bonr.2022.101604 (PMC9304727; doi:10.1016/j.bonr.2022.101604)
Supplement: Supplementary file 1 — Supplementary material [file mmc1.docx]

**Supplementary Information**

A pilot study of nanoscale investigation of localized bone tissue near lacuna in fracturing women

Wen Qian^a^, Roman Schmidt^a^, Joseph A Turner^a^, Mohammed P Akhter^b^, Sue P Bare^b^, Joan M Lappe^b^, Robert R Recker^b,*^ akhtermp@creighton.edu

^a^ Department of Mechanical and Materials Engineering, University of Nebraska-Lincoln, Lincoln, NE 68588-0526, United States of America

^b^ Osteoporosis Research Center, Creighton University, Omaha, NE, 68178, United States of America

⁎Corresponding author.

Dazzi outlined the basic theory of signal detected in AFM-IR [1].

**Absorption of IR Light by the Sample.** Spectroscopy exploits interactions of a material with an incident wave as a function of wavelength (or equivalently, energy or frequency). In the mid-IR range (500 to 4000 cm^-1^), the energy of electromagnetic waves usually corresponds to molecular vibrations. From an optical point of view, materials are characterized by their complex optical index:
$\tilde{n}\left( \lambda\right)=n\left( \lambda\right)+ⅈk\left( \lambda\right)$ (1)

where *λ* is the wavelength, *n*(*λ*) is the real refractive index, and *κ*(*λ*), the imaginary component of the index, is the extinction coefficient.

**Generation of Heat from IR Absorption and Resulting Temperature Rise**. The absorption of the light by a material leads to an increase in its temperature. The evolution of this heat can be easily described by Fourier’s Law:

$\rho C\frac{\partial T}{\partial t}-k\Delta T=\frac{Q(t)}{V}$ (2)

where *ρ* is the density, *C* the heat capacity, *k* the heat conductivity, *V* the volume, *Q*(*t*) is the absorbed heat, and *∆* the Laplace operator.

When the laser pulse is shorter than the sample relaxation time because of photothermal absorption, the temperature can be expressed as:

$T\left( t \right)=T_{max}\frac{t}{t_{p}} for 0\leq t\leq t_{p}$ (3)

$T\left( t \right)=T_{max}exp\left( \frac{t-t_{p}}{\tau_{relax}} \right) for t\geq t_{p}$ (4)

With this theoretical approach, it is easy to understand why the estimated increase of temperature (or *T*_max_) is also a way to measure the absorbance of a sample. All physical phenomena are linear, allowing us to keep the proportionality. The maximum increase in temperature is proportional to the power absorbed by the sample, which is proportional to the absorbance.

**Sample Thermal Expansion.** When the temperature increases in the body of a material, this leads to an increase in the internal stress, resulting in thermal expansion. The temperature field inside the sample is equivalent to a force field, resulting in deformations that depend on the thermomechanical properties of the sample. An expression that links displacement as a function of the temperature is:

$\left( 1-2v \right)\nabla^{2}\mathbf{u}+\nabla\left( \nabla\times\mathbf{u} \right)=2\left( 1+v \right)\alpha_{T}\nabla T$ (5)
where *ν* is Poisson’s ratio, **u** is the displacement vector, *T* is temperature, and *α*_T_ the thermal expansion coefficient.

**Excitation of Cantilever Resonances from the Thermal Expansion.** The AFM–IR technique measures the thermal expansion resulting from IR absorption by using the tip of an atomic force microscope probe. The rapid thermal expansion creates a force impulse on the tip that results in oscillation of the
cantilever at its contact resonant frequencies. The Euler–Bernoulli beam equation is used to estimate the reaction of the cantilever when the expansion occurs under the tip. This equation is given by:

$EI\frac{\partial^{4}z}{\partial x^{4}}+\rho S\frac{\partial^{2}z}{\partial t^{2}}+\gamma\frac{\partial z}{\partial t}=W(x,t)$ (6)

where *E* is the cantilever Young’s modulus, *I* the area moment of inertia, *ρ* the density, *S* the cross-sectional area, *γ* the damping, and *W* the external mechanical source of motion. The cross–sectional area is *S* = *we* and the area moment of inertia I = (1/12)*we^3^* in the case of a rectangular beam, where *w* and *e* are the width and thickness, respectively.

As a result, the general eigenvalue equation for the AFM–IR configuration is given by

$-1+\cos\left( x \right)\cosh\left( x \right)-Ux(\sin\left( x \right)\cosh\left( x \right)-\cos\left( x \right)\sinh\left( x \right))=0$. (7)

The spatial distribution of mode n in the case of contact resonance is described by:

$g_{n}\left( x \right)=\left[ \cos\left( \beta_{n}x \right)-\cosh\left( \beta_{n}x \right) \right]-\left[ \frac{\cos\left( \beta_{n}L \right)-\cosh\left( \beta_{n}L \right)}{\sin\left( \beta_{n}L \right)-\sinh\left( \beta_{n}L \right)} \right]\left[ \sin\left( \beta_{n}x \right)-\sinh\left( \beta_{n}x \right) \right]$. (8)

In our particular case, the cantilever is excited by a source of motion that is the thermal expansion. The corresponding equation of motion can be written as:

$EI\frac{\partial^{4}z}{\partial x^{4}}+\rho S\frac{\partial^{2}z}{\partial t^{2}}+\gamma\frac{\partial z}{\partial t}=W(x,t)$ (9)

where *W(x,t)* is the mechanical source of motion, and where the general solution can be expanded as a sum over eigenmodes:
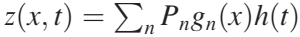
, where *P*_n_ is the amplitude coefficient of the mode *n*.

Oliver and Pharr [2] derived the following relationship for force-displacement curves obtained with an indenter of revolution pressed into an isotropic elastic material as

$S\left( h_{max} \right)=\frac{dp}{dh}\left( h_{max} \right)=\frac{2}{\sqrt{\pi}}E_{r}\sqrt{A_{c}\left( h_{max} \right)}$ (10)

This relationship has been shown to be a good approximation for a Berkovich indenter tip [3]. Here, *P* represents the applied load. *S*(*h*_max_) is the derivative of the unloading curve at the point of initial unloading, *h*_max_, which is determined by fitting 40%–95% of the unloading curve. *A_c_*(*h*) is the contact area over which the material and the indenter are in instantaneous contact. The latter function is determined by a calibration procedure described by Oliver and Pharr. The reduced modulus, *E_r_*, depends on the deformation of the material and the diamond tip as well. According to Hertz, it consists of the sum of two contributions [4]:

$\frac{1}{E_{r}}=\frac{1-v_{specimen}^{2}}{E_{specimen}}+\frac{1-v_{tip}^{2}}{E_{tip}}$ (11)

The indentation modulus: can be calculated with the reduced modulus and the elastic properties of the diamond indenter tip, *v*_tip_ =0.07 and *E*_tip_ =1140 GPa.

$E_{ind}=\left( \frac{1}{E_{r}}-\frac{1-v_{tip}^{2}}{E_{tip}} \right)^{-1}$ (12)

This variable represents: a combination of the local Young’s modulus, *E*_specimen_, and the local Poisson’s ratio *ν* of bone is generally assumed to be 0.3, *V*_specimen_, whereby the material is assumed to be isotropic.

$E_{ind}=\frac{E_{specimen}}{1-v_{specimen}^{2}}$ (13)

The calibration of the device was performed using fused silica with *E*_ind_ =74.4± 2.2 GPa, and the determined area function was validated with a polycarbonate sample with *E*_ind_ =3.4± 0.26 GPa. The nanoindenter was therefore calibrated with two materials that frame the properties of bone in terms of elastic modulus, viscoelasticity, and time-dependent plasticity. For this study, which includes 550 indents, the tip calibration procedure was performed four times. The classical hardness property represents the mean pressure under the tip at maximum load *P*(*h*_max_):

$H=\frac{P\left( h_{max} \right)}{A_{c}\left( h_{max} \right)}$ (14)

**Captions:**

Table S1: A total of nine data points around a lacuna using force-control indentation (a Case sample).

Figure S1: Localized IR spectrum of “lacunae-near” and “lacunae-far” for three samples, representing: (a) 17 separate spectrums around a lacuna for Case sample A2519; (b) 16 separate spectrums around a lacuna for Case sample A2519; (c) 18 separate spectrums around a lacuna for Control sample A2531..

**Figures and Tables**

Table S1: A total of nine data points around a lacuna using force-control indentation (a Case sample).

| **File** | **Hc**  **(nm)** | **Pmax**  **(µN)** | **S**  **(µN/nm** | **A**  **(nm^2)** | **Hmax**  **(nm)** | **Heff**  **(nm)** | **Er**  **(GPa)** | **H**  **(GPa)** | **A** | **Hf**  **(nm)** |
| --- | --- | --- | --- | --- | --- | --- | --- | --- | --- | --- |
| **nine indents_000 LC.hys** | **1033.90** | **5998.52** | **8.55** | **2.62E+07** | **1546.94** | **1559.97** | **1.48** | **0.23** | **0.20** | **520.97** |
| **nine indents_001 LC.hys** | **1062.27** | **5998.61** | **8.56** | **2.76E+07** | **1574.93** | **1588.05** | **1.44** | **0.22** | **0.29** | **580.21** |
| **nine indents_002 LC.hys** | **1084.72** | **5998.64** | **8.46** | **2.88E+07** | **1602.70** | **1616.40** | **1.40** | **0.21** | **0.30** | **603.61** |
| **nine indents_003 LC.hys** | **992.56** | **5998.65** | **8.58** | **2.41E+07** | **1503.33** | **1516.67** | **1.55** | **0.25** | **0.38** | **536.13** |
| **nine indents_004 LC.hys** | **1014.88** | **5998.54** | **8.55** | **2.52E+07** | **1527.56** | **1540.76** | **1.51** | **0.24** | **0.35** | **549.51** |
| **nine indents_005 LC.hys** | **1068.78** | **5998.80** | **8.57** | **2.80E+07** | **1580.64** | **1593.62** | **1.44** | **0.21** | **0.28** | **584.24** |
| **nine indents_006 LC.hys** | **1066.67** | **5998.39** | **8.65** | **2.79E+07** | **1573.50** | **1586.52** | **1.45** | **0.22** | **0.38** | **612.30** |
| **nine indents_007 LC.hys** | **1042.51** | **5998.61** | **8.59** | **2.66E+07** | **1553.11** | **1566.16** | **1.48** | **0.23** | **0.36** | **580.33** |
| **nine indents_008 LC.hys** | **996.32** | **5998.61** | **8.76** | **2.43E+07** | **1497.16** | **1509.97** | **1.57** | **0.25** | **0.29** | **522.59** |


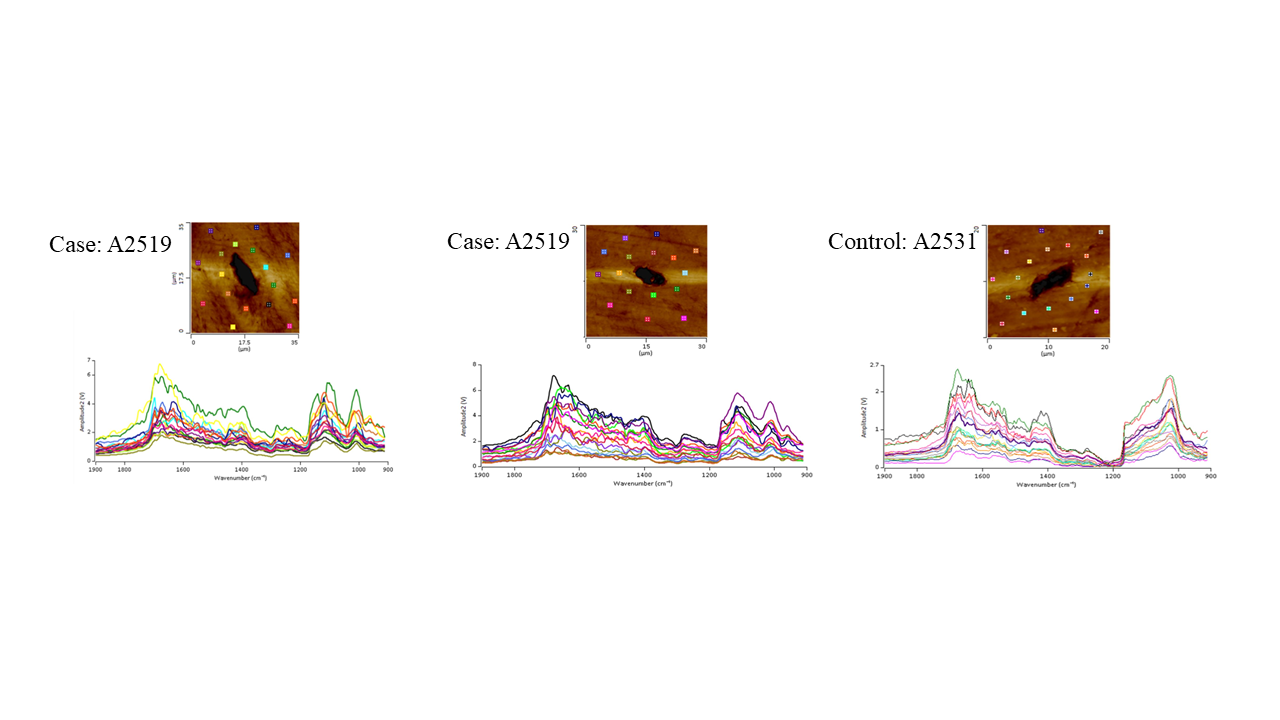


**a**

**b**

**c**

**Figure S1** Localized IR spectrum of “lacunae-near” and “lacunae-far” for three samples, representing: (a) 17 separate spectrums around a lacuna for Case sample A2519; (b) 16 separate spectrums around a lacuna for Case sample A2519; (c) 18 separate spectrums around a lacuna for Control sample A2531..

**References**

[1] Dazzi, A.; Prater, C. B.; Hu, Q.; Chase, D. B.; Rabolt, J. F.; Marcott, C., AFM–IR: Combining Atomic Force Microscopy and Infrared Spectroscopy for Nanoscale Chemical Characterization. *Applied Spectroscopy* **2012,** *66* (12), 1365-1384.

[2] Oliver, W. C.; Pharr, G. M., An improved technique for determining hardness and elastic modulus using load and displacement sensing indentation experiments. *Journal of Materials Research* **1992,** *7* (6), 1564-1583.

[3] Pharr, G. M.; Oliver, W. C.; Brotzen, F. R., On the generality of the relationship among contact stiffness, contact area, and elastic modulus during indentation. *Journal of Materials Research* **1992,** *7* (3), 613-617.

[4] Johnson, K. L, Contact Mechanics. Cambridge, UK: Cambridge University; 84–106; 1985.
